# Population-Based Study of Emergence and Spread of *Escherichia coli* Producing OXA-48–Like Carbapenemases, Israel, 2007–2023

**DOI:** 10.3201/eid3101.240722

**Published:** 2025-01

**Authors:** Elizabeth Temkin, Moshe Bechor, Mor N. Lurie-Weinberger, Alona Keren-Paz, Dafna Chen, Carmela Lugassy, Ester Solter, Mitchell J. Schwaber, Yehuda Carmeli, CPE Working Group

**Affiliations:** National Institute for Antibiotic Resistance and Infection Control, Tel Aviv, Israel (E. Temkin, M. Bechor, M.N. Lurie-Weinberger, A. Keren-Paz, D. Chen, C. Lugassy, E. Solter, M.J. Schwaber, Y. Carmeli); Tel Aviv University Faculty of Medical and Health Sciences, Tel Aviv (M.J. Schwaber, Y. Carmeli)

**Keywords:** *Escherichia coli*, carbapenemase, bacteria, antimicrobial resistance, molecular epidemiology, bacteria, bacterial infections, food safety, Israel

## Abstract

*Escherichia coli* producing OXA-48–like carbapenemases (OXA-EC) is considered a high-risk pathogen spread primarily in the community in low- and middle-income countries and nosocomially in high-income countries. We investigated the emergence and spread of OXA-EC in Israel, a high-income country with strong carbapenemase-directed infection control in healthcare institutions, by conducting a population-based study using data and isolates from the national surveillance system. A total of 3,510 incident cases of OXA-EC occurred during 2007–2023. During 2016–2023, annual cases rose from 41 to 1,524 and nonnosocomial cases rose from 39% to 57%. Sixty-three sequenced isolates belonged to 20 sequence types (STs) and had 3 *bla*_OXA_ alleles (*bla*_OXA-244_, *bla*_OXA-48_, and *bla*_OXA-181_); 71% were chromosomally encoded, and 29% were plasmid-encoded. Nosocomially and non–nosocomially acquired isolates belonged to the same STs and alleles. Most outbreaks involved multiple STs and could only partially be explained by plasmid dissemination. Measures for confronting OXA-EC might need reconsideration.

Enterobacterales harboring OXA-48–like carbapenemases are notable for their susceptibility to third-generation cephalosporins and low-level resistance to carbapenems (*1*). Dissemination of *bla*_OXA-48–like_ occurs by both plasmid transfer and clonal spread (*2*). Although *bla*_OXA-48–like_ was initially characterized as exclusively plasmid-borne (*1*), later reports described chromosomally carried *bla*_OXA-48–like_ (*3*). A review published in 2017 reported that *bla*_OXA-48–like_ was extremely rare in the United States but relatively common in Europe and spreading in the Middle East, Africa, Asia, and South America (*4*). In high-income countries, most carbapenemase-producing Enterobacterales (CPE) are either nosocomially acquired or imported from CPE-endemic countries, whereas in low- and middle-income countries, community spread is common (*5*,*6*). However, a recent study of 1 OXA-48–like *Escherichia coli* clone (OXA-244–producing sequence type [ST] 38) in Europe concluded that community transmission was its main mode of spread (*7*).

During 2007–2011, the first 4 cases of OXA-48–like colonization or infection were detected in Israel, all in patients who had been hospitalized in or traveled to Jordan or Georgia (*8*,*9*). In 2012, a total of 57 patients were involved in an outbreak of OXA-48–producing Enterobacterales (OXA-PE) in a neonatal intensive care unit (*10*). Despite a stringent national intervention to limit the spread of CPE in the healthcare system (*11*), we observed a sharp increase in cases of *E. coli* producing OXA-48–like carbapenemases (OXA-EC). In 2008, we predicted a scenario of community spread of a plasmidborne carbapenemase in a common human pathogen (*12*) that appears to be coming true in the form of OXA-EC.

The objective of our study was to describe the spread of OXA-EC in Israel. Specifically, we aimed to determine the incidence of OXA-EC over time and by acquisition source, the proportion of OXA-EC case-patients with no recent history of healthcare exposure (suggesting community transmission), the risk for progression from OXA-EC carriage to bloodstream infection, whether nosocomially acquired and non–nosocomially acquired isolates are related, and whether the OXA-EC epidemic is driven by a single allele, plasmid, or clone. We hypothesized that the increase in OXA-EC incidence stems from community spread of *bla*_OXA-48–like_ through plasmids on multiple *E. coli* clones.

## Methods

### Study Design and Setting

The study was a country-level, population-based descriptive study of OXA-EC in Israel during 2007–2023. Israel has conducted active surveillance with mandatory reporting of carbapenem-resistant Enterobacterales (CRE) since 2007. Patients are screened for CPE carriage upon hospital admission if they are transferred from another healthcare facility or were hospitalized in an acute care hospital or long-term care facility in the previous 6 months. Patients are screened during their hospital stay if they had contact with a newly detected CPE carrier or are transferred from a high-risk ward, or as part of routine screening in high-risk wards.

### Data Sources

We used data from Israel’s national CRE surveillance system, which records all new cases of CPE detected by screening or clinical culture. In addition, since October 2022, we prospectively investigated new CPE acquisitions that were classified as nonnosocomial to determine the possible source; local infection control staff questioned these patients about contact with the healthcare system in the past year and foreign travel. We used the national surveillance system of bloodstream infections (BSIs) caused by sentinel bacteria to determine the incidence of OXA-EC BSI among patients with OXA-EC first detected by screening.

### Definitions

CPE acquisition refers to the first time that CPE with a given carbapenemase was detected in a patient. We classified acquisitions as nosocomial if CPE was detected >48 hours after admission, upon transfer to another healthcare institution, or upon readmission within 30 days. We classified all other acquisitions as nonnosocomial, which includes healthcare-associated cases and imported cases. We defined nosocomial cases as belonging to a ward-level outbreak if >2 cases of nosocomial OXA-EC were detected in the same ward, with <30 days between cases. We defined nosocomial cases as probably hospital-acquired during an outbreak if >2 cases of nosocomial OXA-EC were detected in different wards, with <30 days between cases; we qualified those acquisitions as probable because they might have been cases introduced from the community and detected after the second hospital day.

### Laboratory Methods

We conducted CPE screening by rectal swab. We processed specimens according to national guidelines for CRE testing (*13*). Carbapenemase identification (*bla*_KPC_, *bla*_NDM_, *bla*_OXA-48–like,_ and *bla*_VIM_) by PCR or lateral flow immunoassay has been mandatory since 2016 for all Enterobacterales growing on screening plates or having a meropenem MIC >0.25 μg/mL. In most laboratories in Israel, screening is performed using mSUPERCARBA (CHROMagar, https://www.chromagar.com), which has high sensitivity (*14*). Enrichment is not recommended because it delays results needed quickly to guide infection control measures.

We performed additional analyses on 235 OXA-EC isolates collected during 2021–2023 from 34 healthcare institutions. We conducted isolate identification and antibiotic susceptibility testing by using VITEK 2 (bioMérieux, https://www.biomerieux.com) or the disk diffusion method (for imipenem, ertapenem, and ceftazidime/avibactam) based on Clinical and Laboratory Standards Institute breakpoints (*15*). We determined meropenem MICs by agar dilution. To determine whether the spread of OXA-EC is monoclonal, we performed Fourier-transform infrared spectroscopy (FTIR) by using previously described methods (*16*). FTIR groups isolates into clusters by phenotype; for *E. coli*, the clusters are good approximations of clones identified by genotyping (*17*–*19*). On the basis of the dendrogram, we selected 63 isolates representing large clusters, small clusters, and singletons; different healthcare facilities; and nosocomial and nonnosocomial acquisitions to undergo whole-genome sequencing (WGS). DNA samples were sequenced using Oxford Nanopore at SNPsaurus (Eugene, OR, USA). We assigned STs to isolates on the basis of the Achtman and Pasteur schemes, and we performed core-genome multilocus sequence typing using PubMLST (https://pubmlst.org). We examined whether nosocomially acquired and non–nosocomially acquired isolates belonged to separate clones or clusters. We also examined whether outbreaks were monoclonal or monocluster.

To understand the role of plasmids in OXA-EC spread, we detected plasmids in sequenced isolates by using PlasmidFinder 2.0.1 (https://cge.food.dtu.dk/services/PlasmidFinder). We performed plasmid visualization by using Proksee (https://proksee.ca). To display the relationship between STs, alleles, and plasmids, we constructed a pan-genome tree by using Roary 3.12.0 (https://bioweb.pasteur.fr/packages/pack@Roary@3.12.0), followed by a maximum-likelihood tree built with RAxML 8.2.12 (https://bioweb.pasteur.fr/packages/pack@RAxML@8.2.12) using the general time-reversible plus gamma model.

### Statistical Analysis

We summarized incident cases of OXA-EC occurring during 2007–2023 by using descriptive statistics (percentage or median and interquartile range [IQR]). We plotted an epidemic curve of annual incident cases of Enterobacterales producing OXA-48–like carbapenemases during 2007–2023, stratified by species (*E. coli*, *Klebsiella pneumoniae*, and other Enterobacterales). To determine the incidence of OXA-EC over time and by acquisition source, we plotted annual incident OXA-EC cases from 2016 (the start of mandatory carbapenemase testing) to 2023, stratified by acquisition type as defined previously. 

We used piecewise linear regression to determine whether the change in cases per 1 million population per year differed among 3 periods (2007–2015, 2016–2021, and 2022–2023). We performed a χ^2^ test to determine whether the proportion of cases that were nonnosocomial differed between the periods 2016–2021 and 2022–2023. We used data from our prospective investigations of cases detected during October 2022–December 2023 to determine the proportion of OXA-EC cases in persons with no recent history of healthcare exposure. We then calculated the percentage (and exact 95% CI) of patients with OXA-EC first detected by screening who later had OXA-EC BSI. We excluded patients with <2 days of follow-up. We performed those analyses in Stata 14.2 (https://www.stata.com/stata14).

We used the adjusted Wallace coefficient to calculate concordance between: FTIR and MLST, FTIR and OXA-48–like alleles, and MLST and OXA-48–like alleles. We performed the calculations by using an online calculator (*20*).

### Ethics Considerations

 The study was approved by the Institutional Review Board at Tel Aviv Sourasky Medical Center. The informed consent requirement was waived for this analysis of routinely collected surveillance data.

## Results

### Description of Patients

During 2007–2023, a total of 3,510 incident cases of OXA-EC occurred in Israel. The median age of patients was 67 years (IQR 51–78 years), and the median time from acute care hospital admission to detection of nosocomial acquisition was 10 days (IQR 5–18 days) (Table 1).

**Table 1:** Characteristics of 3,510 OXA-EC carriers, Israel, 2007–2023*

Characteristic	No. (%)
Sex	
M	1,936 (55.1)
F	1,556 (44.3)
Not recorded	18 (0.5)
Site where OXA-EC was first detected	
Rectal screening culture	3,356 (95.6)
Clinical culture	154 (4.4)
Nosocomial acquisition	1,661 (47.3)
Sites of nosocomial acquisition	
Acute care hospital	1,378/1,661 (83.0)
Internal medicine ward	447/1,378 (32.4)
Surgical ward	416/1,378 (30.2)
Intensive care unit	134/1,378 (9.7)
Other	381/1,378 (27.6)
Non–acute care setting	283/1,661 (17.0)
Nursing home	197/283 (69.6)
Post–acute care hospital	76/283 (26.9)
Other	10/283 (3.5)

*OXA-EC, *Escherichia coli* producing OXA-48–like carbapenemases.

### Trends in OXA-EC Incidence

**Figure 1:**
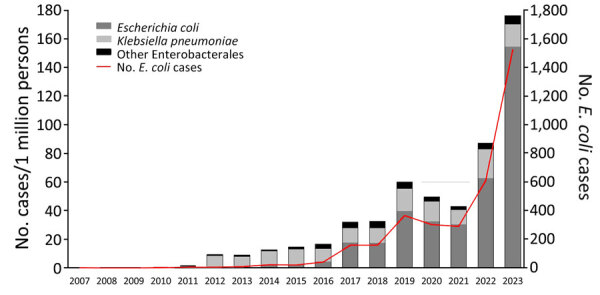
Newly detected cases of Enterobacterales producing OXA-48–like carbapenemases (per 1 million persons) and number of cases of *Escherichia coli* producing OXA-48–like carbapenemases, Israel, 2007–2023. PCR testing for carbapenemases has been required in Israel since 2016, and under-detection of OXA-48-like–producing isolates might have occurred before 2016.

We calculated the annual incidence of *E. coli*, *K. pneumoniae*, and other Enterobacterales producing OXA-48–like carbapenemases (Figure 1). No more than 4 annual cases of OXA-EC occurred until 2013; the number rose to 41 in 2016 and to 1,524 in 2023. During 2007–2015, OXA-EC cases per 1 million population increased by 0.7 (95% CI −0.6 to 2.0) per year, but the increase was not significant. Cases per 1 million population increased by 8.1 (95% CI 6.1–10.2) per year during 2016–2021 and by 101.5 (95% CI 85.6–117.5) per year during 2022–2023. The change in slope between the later 2 periods was significant (93.4 [95% CI 76.4–110.4]). In contrast, cases of *K. pneumoniae* producing OXA-48–like carbapenemases per 1 million population increased by only 1.0 (95% CI 0.1–1.9) per year during 2016–2021 and decreased nonsignificantly by 0.7 (95% CI −7.5 to 6.1) per year during 2022–2023. In both periods, the annual rate of cases of other Enterobacterales producing OXA-48–like carbapenemases did not change significantly. Cases of all species producing OXA-48–like carbapenemases declined during 2020–2021, the years of the SARS-CoV-2 pandemic.

### Place of Acquisition

**Figure 2:**
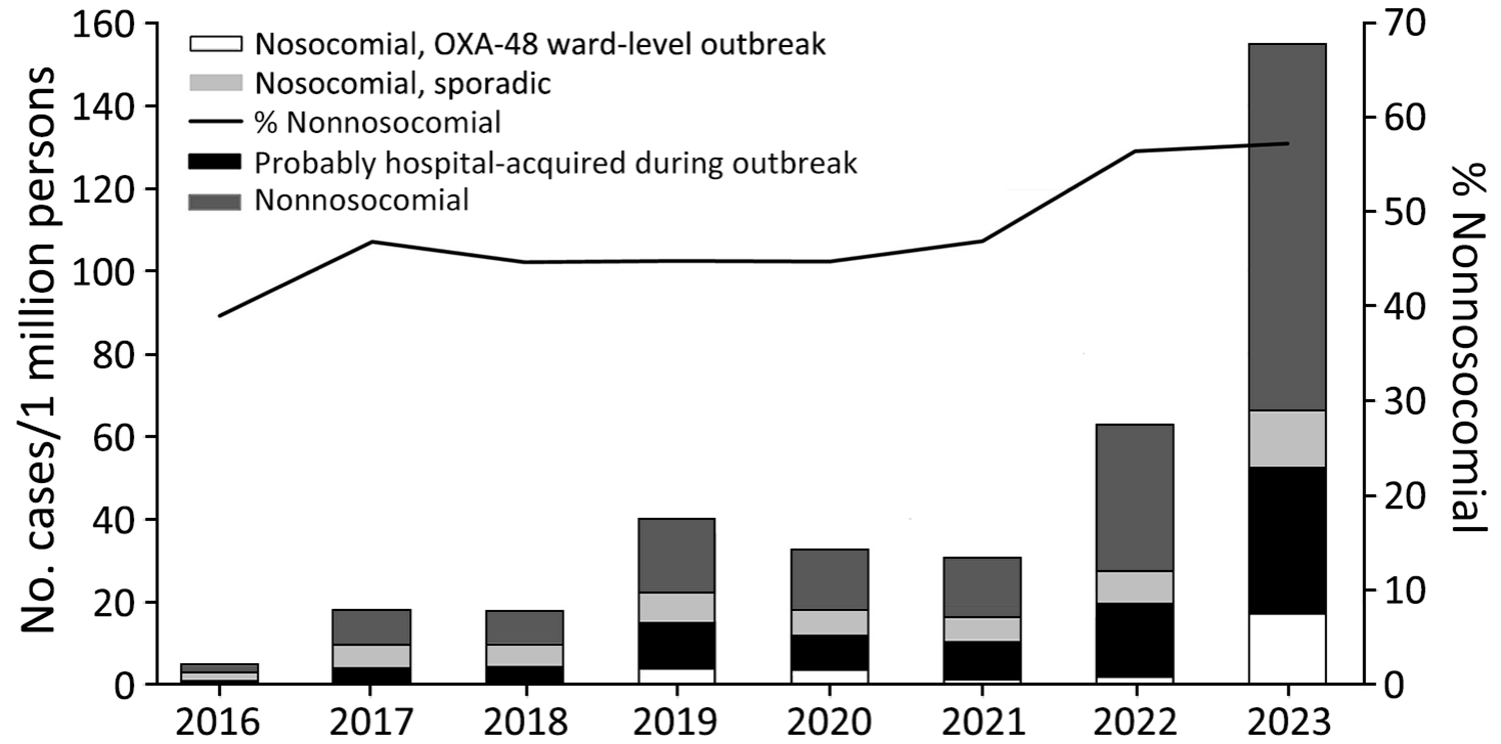
Newly detected cases of *Escherichia coli* producing OXA-48–like carbapenemases, by type of acquisition, Israel, 2016–2023. Nosocomial acquisition is defined as detected >48 hours after admission to hospital or long-term care facility, upon transfer, or upon readmission if previous admission occurred in the last 30 days. Ward-level outbreak is defined as >2 nosocomial cases within the same ward, <30 days between cases. Probably hospital-acquired during an outbreak is defined as >2 nosocomial cases in the same institution but not in the same ward, <30 days between cases. Nonnosocomial category might include healthcare-associated cases.

We calculated the incidence of OXA-EC cases by type of acquisition and by year since 2016 (Figure 2). The percentage of cases that were nonnosocomial was significantly higher during 2022–2023 than during 2016–2021 (57.0% vs. 45.3%; p<0.001). Most nosocomial cases (83.0%) were acquired in acute care hospitals (Table 1).

We analyzed the results of the prospective investigation of sources of OXA-EC acquisition conducted during October 2022–December 2023 (Table 2; Appendix Table 1). Of 1,750 cases, 1,000 (57.1%) were nonnosocomial. Of those, 518 (51.8%) occurred in patients who had contact with the healthcare system in Israel during the previous year, 53 (5.3%) cases were imported, and 429 cases (24.5% of all cases) were classified as community-acquired.

**Table 2:** Source of OXA-EC acquisition among 1,750 carriers, Israel, October 2022–December 2023*

Classification	No. (%)
Initial classification	
Nosocomial†	750 (42.9)
Nonnosocomial	1,000 (57.1)
Reclassification of non-nosocomial after investigation‡	
Healthcare-associated	518 (51.8)
Imported§	53 (5.3)
Community-acquired	429 (42.9)
Community-acquired as % of all cases	429 (24.5)

*OXA-EC, *Escherichia coli* producing OXA-48–like carbapenemases. †Detected >48 hours after hospital admission, upon transfer, or upon readmission within 30 days. ‡Percentages indicate percentages of patients with acquisition initially classified as nonnosocomial. §Includes cases in residents of Israel reporting recent foreign travel, tourists, medical tourists, and foreign workers.

### OXA-EC BSI

Throughout the study period, 41 cases of BSI caused by OXA-EC occurred. Among the 3,356 patients with OXA-EC first detected by screening, 16 later had OXA-EC BSI (0.5% [95% CI 0.3%–0.8%]).

### Susceptibility of OXA-EC

We further characterized 235 OXA-EC isolates stored at the reference laboratory; they comprised 10% of new cases detected during 2021–2023. A total of 220 (93.6%) isolates were susceptible to meropenem, 44.0% to ceftazidime, 43.2% to ceftriaxone, and 96.2% to ceftazidime/avibactam (Appendix Table 2). Of the meropenem-susceptible isolates, 170 (77.3%) had an MIC <0.25.

### Clonal Analysis of OXA-EC

A dendrogram generated by FTIR (Appendix Figure 1) shows that OXA-EC isolates belong to multiple clusters. The 235 isolates formed 59 singletons, 25 small clusters (2–5 isolates), and 7 large clusters (>6 isolates). Large clusters accounted for 106 (45.1%) isolates. We selected 63 representative isolates for WGS. They comprised 20 STs based on the Achtman scheme and 14 STs based on the Pasteur scheme.

### Association between Clusters/STs and Acquisition Site

Of 19 FTIR clusters containing >3 isolates, 15 contained both nosocomial and non-nosocomial isolates (including those classified as community-acquired). Likewise, we found both nosocomial and nonnosocomial isolates in all 5 of the Achtman STs that included isolates from >3 unique patients.

### Clonality of Outbreaks

The dendrogram includes 100 isolates from patients with nosocomial OXA-EC acquired during a ward-level or hospital-wide outbreak. No outbreaks for which we tested >1 isolate were monocluster or monoclonal. For example, the 22 isolates from an outbreak at hospital E during January–August 2023 comprised 1 dominant cluster (no. 371) and 5 singletons (and >3 STs). Conversely, clusters and STs were not hospital-specific; the largest cluster (no. 381) contained 28 isolates acquired in 6 hospitals and outside the hospital.

### Allele Distribution

WGS revealed that OXA-EC belonged to 3 *bla*_OXA-48–like_ alleles: *bla*_OXA-244_ (n = 36, 57.1%), *bla*_OXA-48_ (n = 17, 27.0%), and *bla*_OXA-181_ (n = 10, 15.9%). All 3 alleles were present in both nosocomial and nonnosocomial isolates (including isolates classified as community-acquired).

### Concordance between FTIR and WGS Results

Concordance between FTIR clusters and Achtman STs was high (0.98 [95% CI 0.96–1.00]); all isolates in each FTIR cluster (except for cluster no. 368) belonged to a single ST. We also observed high concordance between FTIR clusters and alleles (0.97 [95% CI 0.94–1.0]), whereas concordance between Achtman STs and alleles was low (0.42 [95% CI 0.29–0.55]). Because ST typing indicates more distant evolutionary origins than FTIR, the difference in concordance suggests that the introduction of the *bla*_OXA-48–like_ alleles into *E. coli* populations is a relatively recent event. This conclusion is also supported by the finding that no allele was associated with only 1 ST.

### Role of Plasmids

We identified and visualized 3 different plasmids carrying *bla*_OXA-48–like_ (Appendix Figures 2–4). The plasmids were found in 29% of the sequenced isolates. The transfer of plasmids could only partially explain the nonclonal spread of OXA-EC. In all 10 OXA-181–producing isolates, *bla*_OXA-181_ was carried on the composite plasmid ColKP3-IncX3. Among the 17 OXA-48–producing isolates, in 3 *bla*_OXA-48_ was carried on the plasmid IncL(pOXA-48) and in 5 *bla*_OXA-48_ was carried on the composite plasmid IncFII(pRSB107)–IncFIA-IncFIB(AP001918); 9 isolates had no plasmid. All isolates encoding the *bla*_OXA-244_ allele had no plasmid. 

### Dissemination of Alleles and Plasmids within STs

**Figure 3:**
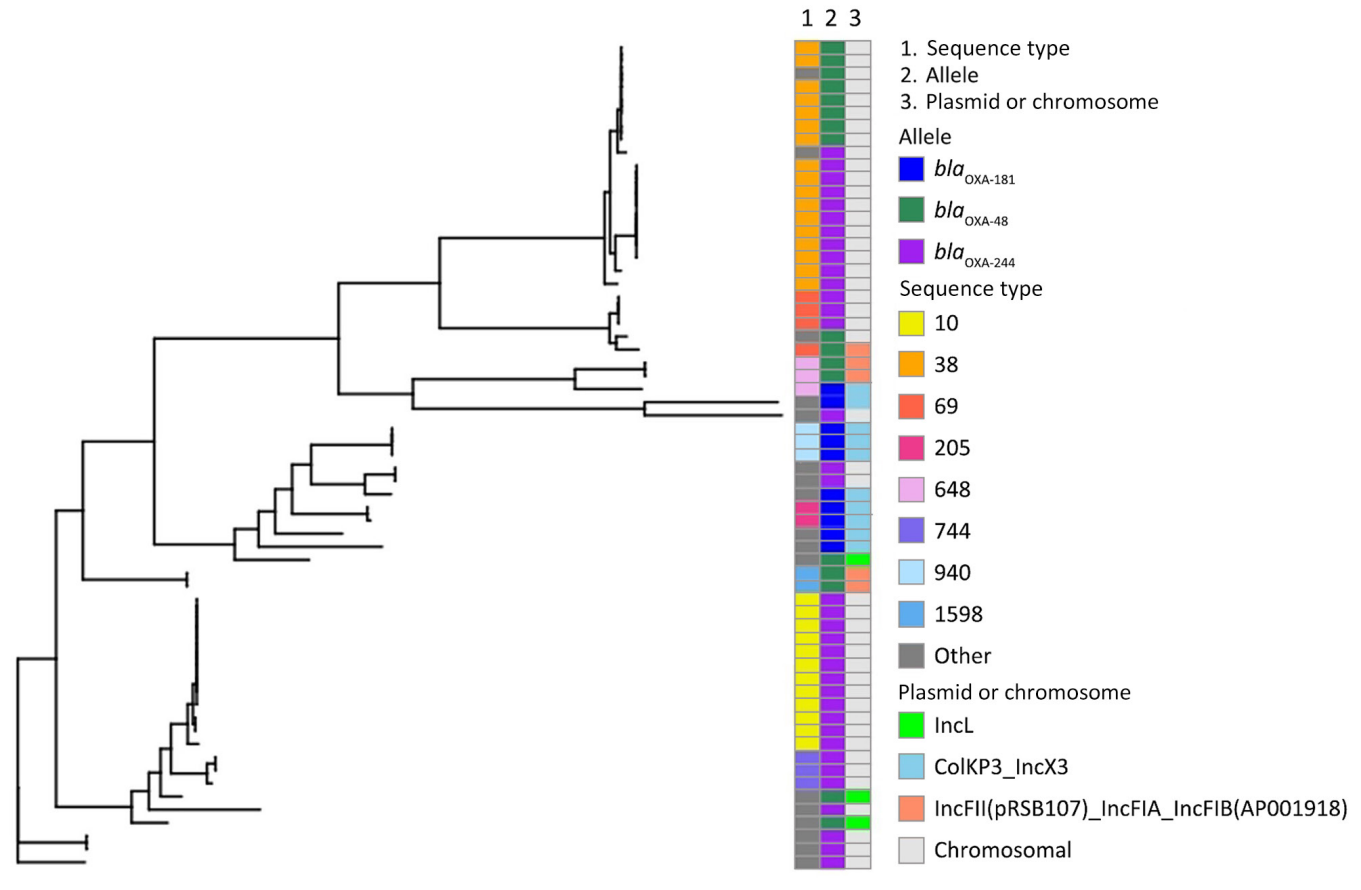
Pangenome phylogenetic tree of 63 isolates of *Escherichia coli* producing OXA-48–like carbapenemases, Israel, 2021–2023.

We examined the relationships among STs, alleles, and plasmids of the sequenced isolates (Figure 3). Alleles and plasmids were scattered among different and distant STs. Only a fraction of allele distribution among STs correlated with plasmid distribution among STs. These results indicate independent outbreaks of the 3 *bla*_OXA-48–like_ alleles, involving multiple STs, each with a different molecular mode of spread: *bla*_OXA-181_ is transmitted through a single plasmid between different STs, *bla*_OXA-48_ spreads by 2 plasmids and by the expansion of ST38 carrying *bla*_OXA-48_ on the chromosome, and *bla*_OXA-244_ is chromosomally encoded and found on 9 STs, suggesting multiple introductions of this gene into the *E. coli* population.

## Discussion

We studied the epidemiology and molecular epidemiology of OXA-EC in Israel during 2007–2023. The incidence of OXA-EC colonization or infection has risen sharply. As we hypothesized, the proportion of cases that are non-nosocomial has risen, comprising more than half of cases during 2022–2023; approximately one quarter were community-acquired. However, the number of hospital-acquired cases also rose, particularly in 2023, probably because of inadequate detection and isolation of imported cases. The risk for OXA-EC bacteremia among OXA-EC carriers was low (0.5%). Most OXA-EC isolates (93.6%) were susceptible to meropenem, and 44.0% were susceptible to ceftazidime. The spread of OXA-EC represents parallel dissemination of 3 different *bla*_OXA-48–like_ alleles occurring in multiple clusters and STs. Nosocomial OXA-EC outbreaks often included >1 clone, which might indicate either detection of unrelated OXA-EC carriers who might have introduced the strain from the community or transmission of the *bla*_OXA-like_ gene between clones through mobile elements. Contrary to our hypothesis that ongoing plasmid spread of *bla*_OXA-48–like_ to multiple *E. coli* clones caused the rise in incidence, we found that *bla*_OXA-48–like_ was not carried on plasmids in more than two thirds of sequenced isolates. Reports on the proportion of OXA-PE that is community-acquired are scarce (*21*,*22*). A scoping review of CPE in the community noted that most studies do not distinguish between infection or colonization that is community-acquired versus community-onset (which might be healthcare associated) (*23*). A survey from 2020–2021 examining OXA-244–producing *E. coli* ST38 in Europe determined that community transmission was the main mode of spread; evidence included the high proportion of isolates from outpatient urine samples and the geographic dispersion of cases within countries (*7*). The authors suggested that transmission might be foodborne.

As in previous studies (*3*,*24*), we found that *bla*_OXA-244_ is encoded chromosomally, indicating that it was introduced into the *E. coli* population on multiple occasions, integrated into the chromosome, and lost its ability to mobilize to other strains. Thus, in Israel, the spread of OXA-244–producing *E. coli* is by parallel expansion of multiple clones. In contrast, *bla*_OXA-181_ was encoded on a plasmid, and its dissemination was driven by plasmid spread and by the expansion of clones carrying the plasmid. *bla*_OXA-48_ had a mixed mode of spread; 2 different plasmids carried this allele and were found in 47% of the isolates, whereas the allele was chromosomally encoded in 53% of the isolates.

We found that nosocomial outbreaks were polyclonal and that not all polyclonal spread was plasmid driven. Several possible explanations exist for these polyclonal, non–plasmid-driven outbreaks. First, nondominant clusters within an outbreak might indicate cases that were present but not detected upon admission. Second, as the result of introduction and diversification of existing clones, multiple clusters/STs might be spreading simultaneously in a single institution, as researchers have described in multidrug-resistant *Acinetobacter baumannii* (*25*). Third, mobile genetic elements other than plasmids might transfer *bla*_OXA-48–like_ between clones.

The rising incidence of OXA-EC indicates that the measures that contained Israel’s outbreak of *K. pneumoniae* carbapenemase–producing *K. pneumoniae* (screening, isolation, cohorting, and staff cohorting) (*11*) are not sufficient to control OXA-EC. Possible reasons for this failure are, first, unidentified community sources of OXA-EC, such as livestock, wildlife, and fresh vegetables (*26*,*27*); such sources are unaffected by hospital-based infection control measures. Second, community acquisition of OXA-EC means that the healthcare-focused criteria for screening at hospital admission are inadequate, leading to in-hospital spread by carriers who are undetected and not isolated. Third, when screening occurs, the low MICs of most OXA-EC might curtail their detection as CPE, and thus carriers are not isolated. Fourth, reservoirs in the hospital environment, such as mattresses and sinks, might be a source of OXA-PE spread (*26*,*27*). Fifth, although plasmids were not the dominant mode of transmission of sequenced OXA-EC in our study, traditional CPE control measures are less effective in limiting plasmid-mediated nosocomial transmission than clonal spread (*28*).

Our findings raise policy questions regarding OXA-EC control efforts in hospitals. Most OXA-EC are carbapenemase-producing but not carbapenem-resistant. Given the low risk for severe infection among carriers, the availability of treatment options, and substantial spread outside of the hospital, the question is whether targeting of infection control efforts toward OXA-EC should be stopped. The benefit would be a sharp decrease in the number of patients requiring epidemiologic investigation and isolation. The risk is that nosocomial transmission will probably accelerate, creating a growing reservoir of carriers that would eventually translate into a higher number of clinical infections. Moreover, although we found high in vitro susceptibility to meropenem and moderate-to-high in vitro susceptibility to third-generation cephalosporins and aminoglycosides, a gap exists between in vitro and in vivo success; some studies have reported high rates of clinical failure and death among patients with severe infections caused by OXA-PE (*7*,*29*).

One limitation of our study is that PCR for *bla*_OXA-48–like_ was not universally used before 2016, probably leading to undercounting of cases in earlier years. In addition, the indications for CPE screening in the hospital might be too narrow to identify community-acquired OXA-EC cases, also leading to undercounting in later years. Moreover, nonnosocomial cases might have been misclassified as nosocomial because they were not detected at hospital admission. Further, FTIR and WGS were performed only on a fraction of isolates; additional clusters, STs, alleles, and plasmids might have gone undetected.

In conclusion, the emergence and rise of OXA-EC is concerning and challenges well-established strategies of CRE control. Infection control policymakers should consider the option of demoting OXA-EC from its status as a high-risk pathogen. However, any loosening of restrictions on OXA-EC carriers must be accompanied by monitoring for unintended consequences.

Appendix Additional information about population-based study of emergence and spread of *Escherichia coli* producing OXA-48–like carbapenemases, Israel, 2007–2023.
