# Supplementary material for: Population-Based Study of Emergence and Spread of Escherichia coli Producing OXA-48–Like Carbapenemases, Israel, 2007–2023
Source: Emerg Infect Dis. 2025 Jan;31(1):66–74. doi: 10.3201/eid3101.240722 (PMC11682801; doi:10.3201/eid3101.240722)
Supplement: Appendix — Additional information about population-based study of emergence and spread of Escherichia coli producing OXA-48–like carbapenemases, Israel, 2007–2023. [file 24-0722-Techapp-s1.pdf]

Article DOI: https://doi.org/10.3201/eid3101.240722

EID cannot ensure accessibility for supplementary materials supplied by authors. Readers who have difficulty accessing supplementary content should contact the authors for assistance.

Population-Based Study of Emergence and Spread of *Escherichia coli* Producing OXA-48–Like Carbapenemases, Israel, 2007–2023

Appendix

Appendix Table 1. Source of acquisition of *E. coli* producing OXA-48-like carbapenemases, October 2022–December 2023, detailed

Source	N = 1,750<br>No. (%)
Initial classification	
Nosocomial[1]	750 (42.9)
Non-nosocomial	1,000 (57.1)
Reclassification of non-nosocomial after investigation[2]	
Healthcare-associated	
Hospitalization <48 h in past 6 mo	107 (10.7)
Hospitalization in past 2-up to 4 mo	185 (18.5)
Hospitalization in past 4-up to 6 mo	82 (8.2)
Hospitalization in past 6-up to 12 mo	95 (9.5)
Outpatient treatment in hospital or dialysis in past 6 mo	31 (3.1)
Endoscopy or ambulatory surgery in past 6 mo	18 (1.8)
Imported[3]	53 (5.3)
Community-acquired	429 (42.9)
Final classification as community-acquired, as % of all cases	429 (24.5)

[1]Nosocomial: detected >48 h after hospital admission, upon transfer, or upon readmission within 30 d.
[2]Percentages indicate percentages of patients with acquisition initially classified as non-nosocomial.
[3]Includes cases in residents of Israel reporting recent foreign travel, tourists, medical tourists, and foreign workers.

Appendix Table 2. Antibiotic susceptibility of *E. coli* producing OXA-48-like carbapenemases*

Antibiotic	No. tested	% susceptible
Ceftazidime	234	44.0
Ceftriaxone	234	43.2
Ciprofloxacin	234	32.1
Gentamicin	234	75.2
Amikacin	231	98.7
Sulfamethoxazole/trimethoprim	234	33.8
Meropenem	235	93.6
Ertapenem	235	20.4
Imipenem	235	44.7
Ceftazidime/avibactam	235	96.2

*Method was VITEK 2 automated system for all antibiotics except meropenem, which was tested by agar dilution, and ertapenem, imipenem, and ceftazidime/avibactam, which were tested by disk diffusion.

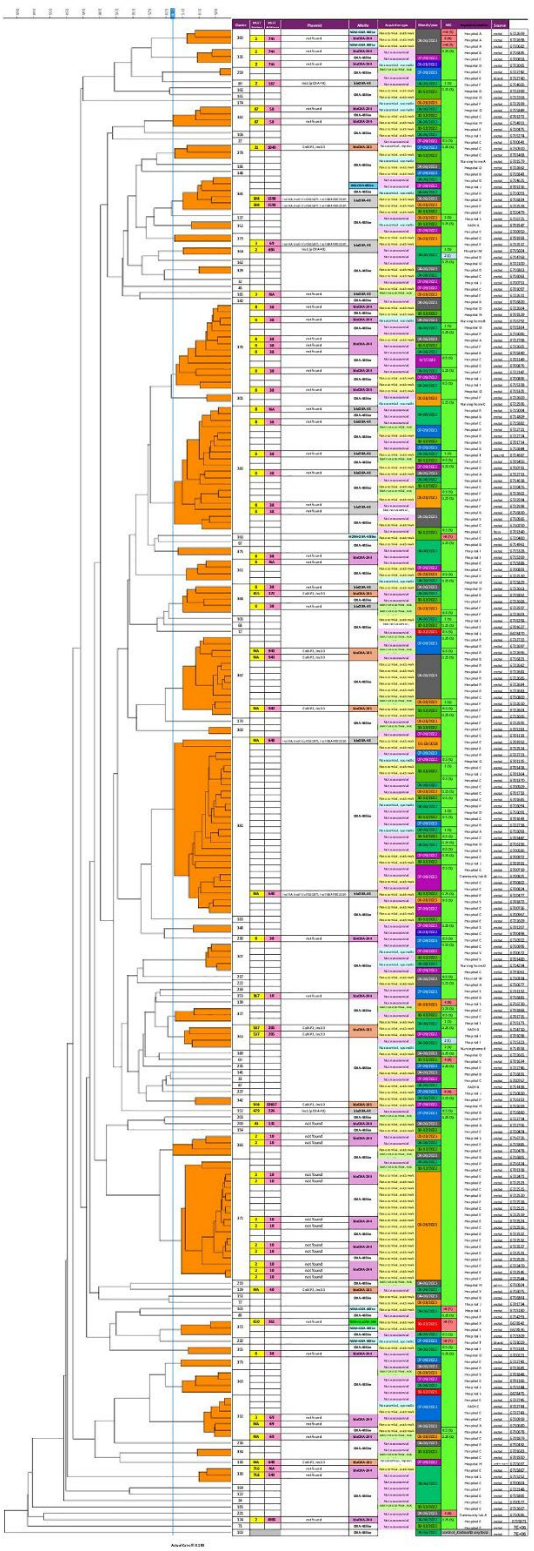

Appendix Figure 1. Dendrogram of *E. coli* producing OXA-48-like carbapenemases acquired in 34 Israeli healthcare institutions, 2021–2023.

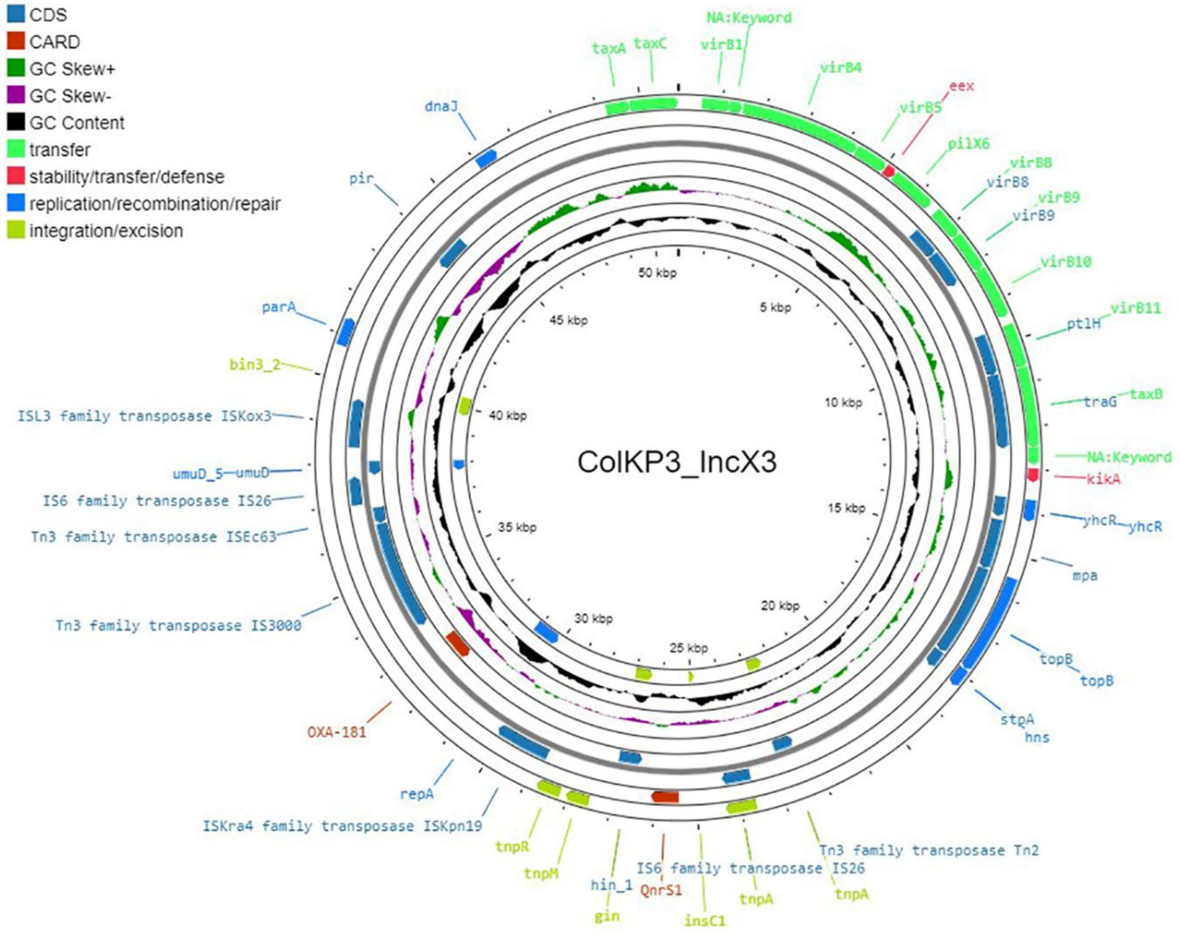

Appendix Figure 2. Composite plasmid ColKP3-IncX3, detected in all 10 sequenced OXA-181-producing *E. coli* isolates.

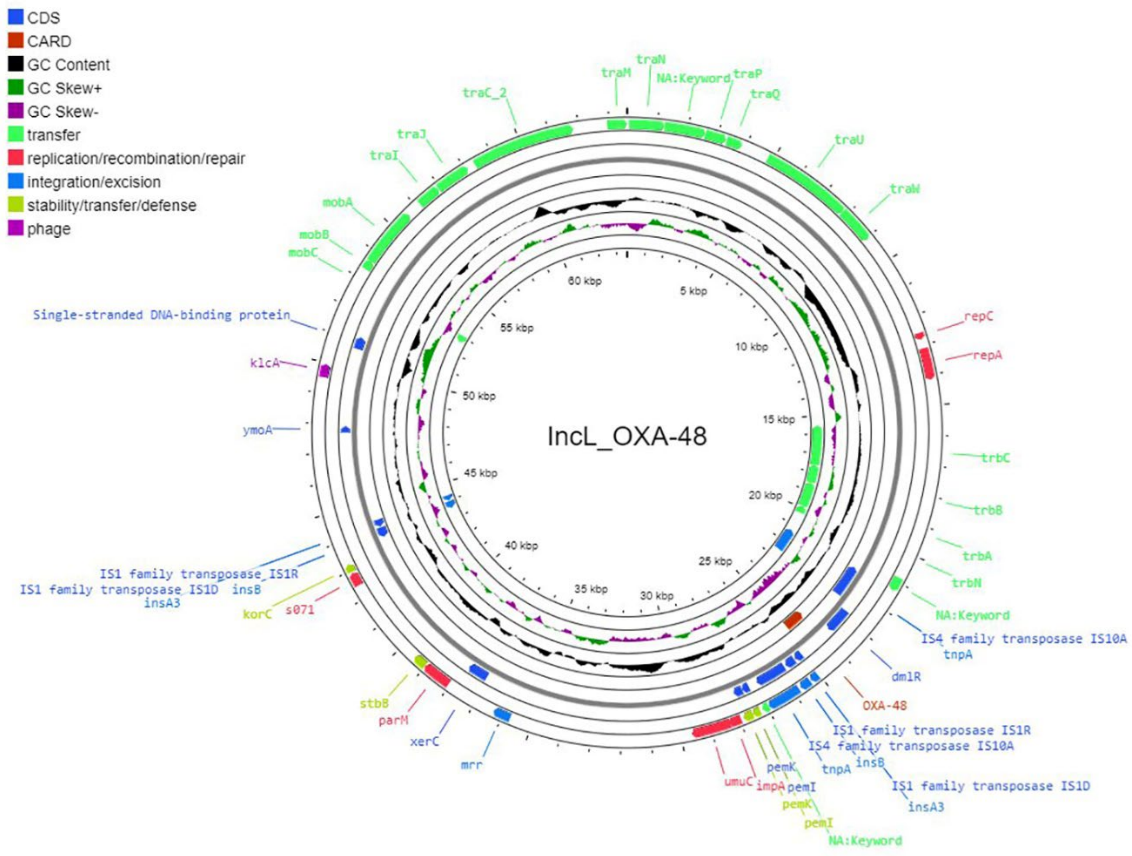

Appendix Figure 3. Composite plasmid IncL(pOXA-48), detected in 3 of 17 sequenced OXA-48-producing *E. coli* isolates.

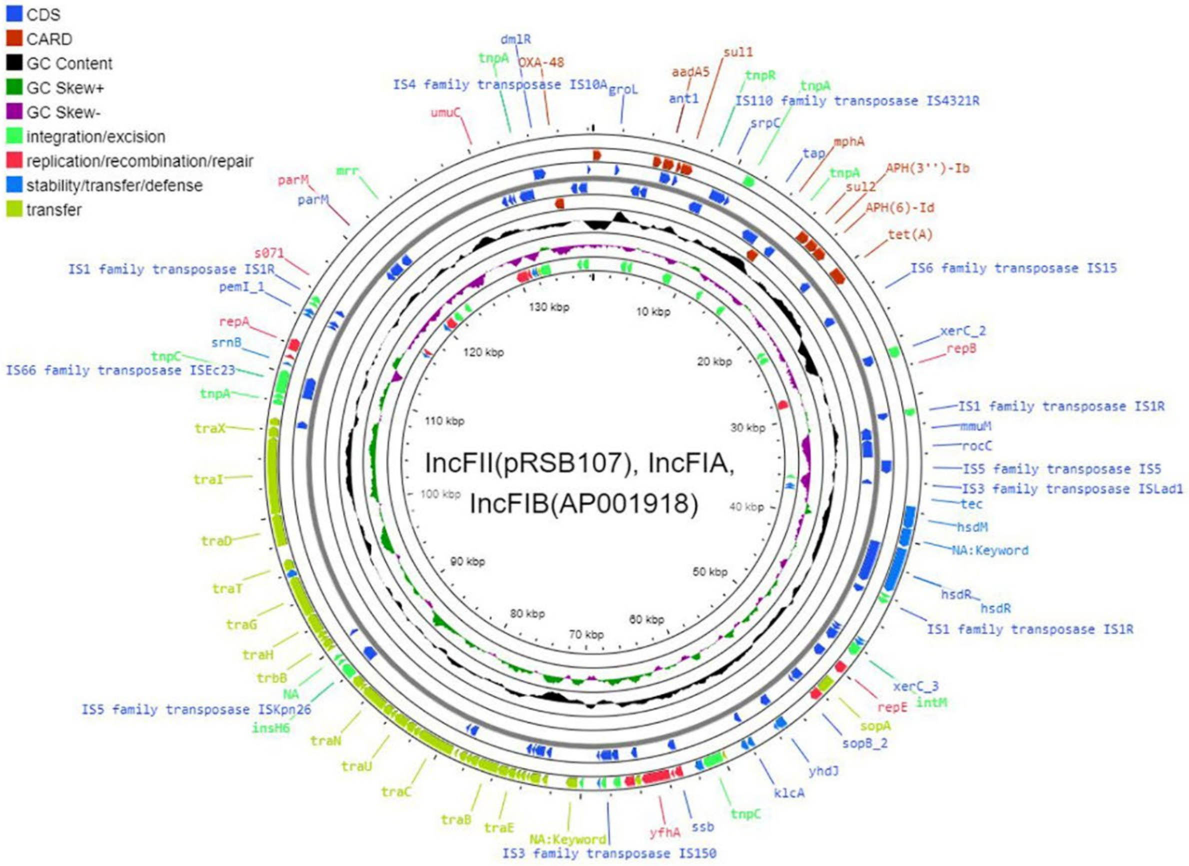

Appendix Figure 4. Composite plasmid IncFII(pRSB107)-IncFIA-IncFIB(AP001918), detected in 5 of 17 sequenced OXA-48-producing *E. coli* isolates.
